# 鞘内合成的肿瘤标志物与血脑屏障完整性在肺癌软脑膜转移诊断中的价值

**DOI:** 10.3779/j.issn.1009-3419.2021.104.08

**Published:** 2021-08-20

**Authors:** 永娟 林, 婷婷 俞, 会颖 李, 震宇 尹, 爱斌 郭

**Affiliations:** 210008 南京，南京大学医学院附属鼓楼医院老年肿瘤科 Department of Geriatric Oncology, Affiliated Nanjing Drum Tower Hospital of Nanjing University Medical School, Nanjing 210008, China

**Keywords:** 肺肿瘤, 软脑膜转移, 脑脊液, 肿瘤标志物, 鞘内合成, 血脑屏障, Lung neoplasms, Leptomeningeal Metastasis, Cerebrospinal fluid, Tumor markers, Intrathecal synthesis, Blood brain barrier

## Abstract

**背景与目的:**

脑脊液(cerebrospinal fluid, CSF)中肿瘤标志物(tumor markers, TM)对软脑膜转移(leptomeningeal metastasis, LM)具有诊断意义，但因TM在CSF中浓度很大程度上取决于血清水平以及血脑屏障(blood brain barrier, BBB)的状态，因此，CSF中TM诊断LM的价值常常被忽视。明确鞘内TM合成、BBB的完整性可为LM临床诊断提供帮助。因此，本研究从BBB完整性和鞘内TM合成角度出发，进一步探讨CSF中TM对肺癌LM诊断的临床价值。

**方法:**

收集2016年12月-2020年3月于南京鼓楼医院治疗的肺癌伴LM的患者25例，另选本院同期治疗的57例非恶性神经系统疾病(nonmalignant neurological diseases, NMNDs)患者为对照组，分析比较BBB完整性和鞘内TM合成的差异，评估LM组CSF中TM检出阳性率与鞘内TM合成的相关性，并动态监测1例LM患者脑室内化疗过程中CSF细胞学、BBB完整性以及鞘内TM合成的变化。

**结果:**

LM患者中94%存在BBB破坏。所有LM患者CSF中至少存在一项TM的鞘内合成，其中1例患者，首次CSF细胞学阴性，但是存在鞘内TM合成，重复腰穿CSF细胞学阳性确诊为LM。LM组CSF中TM检出阳性率与TM鞘内合成不一致。对照组中仅3.5%(2/57)NMNDs患者有BBB破坏，且未在鞘内合成TM，差异有统计学意义(*P* < 0.05)。另外，动态监测CSF细胞学、BBB完整性和CSF鞘内合成的TM与疗效评价一致，且鞘内TM合成的变化早于细胞学。

**结论:**

BBB的评估及鞘内TM合成的分析可为LM患者提供一个早期辅助诊断的工具，动态监测TM可能有助于疗效评估，值得临床推广应用。

## Abstract (en)

**Background and objective:**

Tumor markers (TM) in cerebrospinal fluid (CSF) are useful for diagnosing leptomeningeal metastasis (LM). It has not been fully exploited the diagnostic possibilities of the CSF levels since the basic fact that the TM concentration of CSF depends strongly upon the serum levels as well as upon the condition of the blood brain barrier (BBB). To analyze the intrathecal TM synthesis and evaluate the integrity of BBB can be helpful for the definitive diagnosis of LM. Therefore, the aim of this study was to further explore the clinical value of intrathecal TM synthesis and BBB in the diagnosis for the lung cancer patients with LM.

**Methods:**

Twenty-five lung cancer patients with LM and 57 patients with nonmalignant neurological diseases (NMNDs) admitted to Nanjing Drum Tower Hospital from December 2016 to March 2020 were included. We compared the integrity of BBB and intrathecal TM synthesis between two groups, analyzed the correlation of CSF TM between the detection and intrathecal synthesis, and evaluated serial CSF cytology, the integrity of BBB and intrathecal TM synthesis when intrathecal chemotherapy for one patient.

**Results:**

Ninety-four percent LM patients showed the dysfunction of BBB, and all LM patients showed at least one intrathecal synthesized TM in CSF. In one patient, the CSF cytology was negative for the first time, but LM was eventually diagnosed based on the the intrathecal TM synthesis and positive CSF cytology of repeated lumbar puncture. In LM group, no correlation was observed between the detection and intrathecal synthesized TM in CSF. In the control group, only 3.5% (2/57) NMNDs patients had the dysfunction of BBB and no patients had intrathecal TM synthesis, both the differences of which were statistically significant (*P* < 0.05). Finally, evaluating the CSF cytology, integrity of BBB and intrathecal TM synthesis can be used to assess the intracranial treatment effect. Moreover, intrathecal TM synthesis changes earlier than cytology.

**Conclusion:**

The evaluation of intrathecal TM synthesis and integrity of BBB are novel clinical diagnostic tools. In addition, serial measurement of intrathecal synthesized TM may play an important role in monitoring efficacy of lung cancer patients with LM, which is worthy of further promotion and clinical application.

软脑膜转移(leptomeningeal metastasis, LM)又称为癌性脑膜炎，是晚期肺癌最致命的并发症之一^[1]^，发生率为5%-10%^[2]^，患者预后差，平均生存期仅3.5个月-6个月^[3]^，少数患者生存时间超过12个月^[4]^，尤其是有靶点突变的肺癌患者^[5]^。因此，早期诊断和治疗是改善预后的关键。脑脊液(cerebrospinal fluid, CSF)细胞学检查是诊断LM的金标准，但敏感性仅50%-60%^[6]^。CSF中肿瘤标志物(tumor markers, TM)对LM同样具有诊断作用^[7]^。生理条件下，中枢神经系统(central nervous system, CNS)几乎不生成TM^[8]^。病理条件下，血脑屏障(blood brain barrier, BBB)受到破坏，可使TM从血液进入CSF，或由CNS分泌TM的癌细胞鞘内合成，均可使CSF中TM增高^[9]^，因此，通过检测BBB的完整程度及是否存在鞘内TM合成，可帮助判断CSF中TM的来源，对LM诊断和鉴别诊断具有重要意义。考虑到CSF中TM来源与CNS疾病过程中免疫球蛋白(immunoglobulin G, IgG)生成相似，分子量相近，文献[10]报道CSF和血清TM商值(TM CSF/serum quotients, Q TM)与CSF和血清白蛋白商值(albumin CSF/serum quotients, QA)具有相关性。Corsini等^[11]^通过分析CSF和血清癌胚抗原(carbohydrate antigen, CEA)商值与QA的比值，评估判断CSF中CEA的来源，进一步提高了CSF中CEA在LM辅助诊断的敏感性。因此，本研究入组了肺癌伴LM患者，检测对肺癌诊断敏感性高的肺癌四项，即CEA、糖类抗原125(carbohydrate antigen125, CA125)、细胞角蛋白19片断抗原(cytokeratin 19 fragments, CYFRA21-1)和神经元烯醇化酶(neurone specific enolase, NSE)，通过计算QA评估BBB的完整性、TM指数公式即Q TM/QA评估CNS中鞘内TM的合成，以分析肺癌LM患者CSF中TM来源，旨在提高4种TM在该人群辅助诊断的可行性和敏感性，并进一步讨论TM在肺癌LM疗效评价中的意义。

## 1 材料与方法

### 1.1 研究对象

#### 1.1.1 研究者

本研究回顾性分析于2016年12月-2020年3月入住南京大学附属南京鼓楼医院的肺癌伴LM患者(LM组)，入组标准：①经病理确诊的肺癌患者; ②CSF细胞学找到癌细胞; ③新发的临床系统症状/体征; ④典型的核磁共振(magnetic resonance imaging, MRI)影像学表现; 其中LM的诊断需同时满足条件①，同时满足②、③、④中至少2项。排除标准：①无法获得同期CSF及血清白蛋白水平、TM水平; ②半年内有脑炎、颅脑外伤及脑部放疗史。其中49例诊断为LM，最终入组可获得同期血清和CSF检测指标的患者共25例。LM组所有患者原发病由病理学确诊。治疗上，所有患者前期均经过多种综合治疗方案(包括靶向治疗、多线化疗、免疫治疗等)，LM组均植入Ommaya囊，行经Ommaya囊CSF引流、全身治疗联合脑室内化疗。

#### 1.1.2 对照组

为了进一步评估LM组鞘内合成TM的特异性，选取同期在南京鼓楼医院诊治的57例非恶性神经系统疾病(nonmalignant neurological diseases, NMNDs)患者(NMNDs组)的CSF和血清白蛋白、TM检测值作为对照，所有患者均经腰椎穿刺和影像学检查，排除恶性疾病依据。其中31例(54.4%)为神经系统感染疾病，26例(45.6%)为脑膜瘤。

### 1.2 实验方法

#### 1.2.1 脑脊液相关指标检测

所有LM组、NMNDs组患者均经腰椎穿刺术取CSF，同时抽取外周血行相关指标检测。并记录CSF常规(细胞数)、生化项目指标(如糖、氯化物、白蛋白水平)。同时收集患者CSF和外周血清，行肺癌指标CEA、CA125、CYFRA21-1、NSE检测。所有标本在采集2 h内完成检测项目。其中评估CSF中TM检出情况时，CSF中TM阳性界值参考前期研究中阳性值：即CEA > 6.67 ng/mL，CA125 > 33.44 U/mL，CYFRA21-1 > 4.22 ng/mL，NSE > 13.03 ng/mL^[12]^。

#### 1.2.2 白蛋白商值计算公式及结果判定

利用QA评估BBB完整性功能，即CSF白蛋白与血清白蛋白比率，其中≤7×10^-3^为正常; 介于7×10^-3^-14×10^-3^之间为轻度损伤; 介于14×10^-3^-30×10^-3^为中度损伤; 介于30×10^-3^-100×10^-3^为重度损伤; > 100×10^-3^为完全破裂^[13]^。

#### 1.2.3 评估CNS中TM的鞘内合成

神经系统利用IgG指数评估鞘内合成IgG，其中IgG指数 > 0.7提示鞘内合成了IgG^[10]^。本研究中将CSF中TM类比成IgG，计算TM指数判断是否存在CSN鞘内合成。Q TM即CSF/血清TM，而TM指数公式为：Q TM/QA。其中TM指数 > 0.7，提示CSF中有TM的鞘内合成，最后，通过TM指数定量CSF中TM哪些比例来源于鞘内生成，公式如下^[8]^：

TM鞘内=TMCSF-0.7×QA×TMserum，并计算TM鞘内与TMCSF百分比。

#### 1.2.4 脑脊液中TM动态监测

经Ommaya囊引流CSF是LM一种局部治疗的手段，3例(12%)患者颅高压明显时予引流CSF减轻颅高压症状。所有患者植入Ommaya囊后行周期性脑室内化疗，每次在接受脑室内化疗前留取脑室内CSF，完善CSF常规、生化全体、细胞学及TM等指标检测，评估疗效。重要步骤：常规消毒皮肤，用头皮针插入Ommaya囊内，抽出并丢弃囊和管内3 mL左右CSF，再缓慢收集5 mL-7 mL的CSF行相关指标检测，脑室内化疗后再次予局部消毒，纱布覆盖。

### 1.3 统计学处理

应用SPSS 22.0软件行统计学分析，符合正态分布资料计算均数和标准差，采用均数±标准差(Mean±SD)表示; 计数资料采用百分数表示，两组间比较应用方差分析。CSF中TM指数与TM检出阳性率之间的一致性分析采用Kappa检验，其中Kappa值< 0.2表示二者诊断一致性差，0.21-0.40表示二者诊断一致性弱，0.41-0.60表示二者诊断一致性中等，0.61-0.80表示二者诊断一致性较强，0.81-1.00表示二者诊断几乎相同，*P* < 0.05为差异具有统计学意义。

## 2 结果

### 2.1 LM组与NMNDs组相关指标的比较

本实验组共纳入49例，根据排除标准，最后纳入LM组共25例，详细筛选流程见图 1，均已签署相关知情同意书。LM组男性8例，女性17例，年龄为39岁-62岁，平均(52.04±6.61)岁。NMNDs组男性32例，女性25例，年龄36岁-71岁，平均(45.91±6.12)岁。两组间一般资料无显著差异，性别、年龄、吸烟史、卡氏体能状态(Karnofsky performance status, KPS)评分等相关指标均无统计学差异(*P* > 0.05)，详细资料见表 1。

**图 1:**
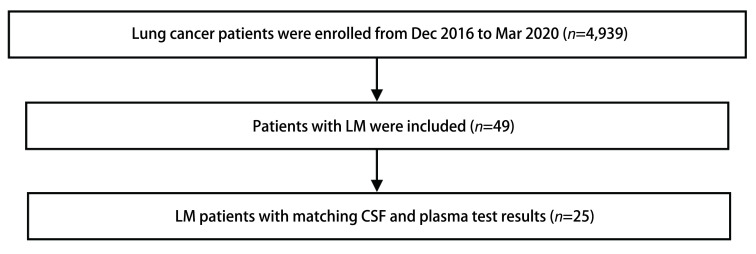
研究筛选流程图 Study subjects. LM: leptomeningeal metastasis; CSF: cerebrospinal fluid.

**表 1:** 一般资料 Baseline characteristics

Category	LM (*n*=25)	NMNDs (*n*=57)	*P*
Gender (Male/Female)	8/17	32/25	0.519
Age (Range, yr)	52.04 (39-62)	45.91 (36-71)	0.591
History of smoking			0.696
Previous or current smoking	7	19	
No smoking	18	38	
Mean KPS (Range)^*^	68.7 (30-90)	71.3 (40-90)	0.105
< 70	9	21	
≥70	16	36	
Clinical symptoms			
Headache/Nausea/Vomiting	15	021	0.549
Focal limb weakness	14	12	0.732
Cranial nerve abnormalities	10	9	0.824
Back pain	3	1	0.196
Urine incontinence	4	0	0.115
Seizure	7	18	0.226
Intracranial hypertension (> 180 cmH_2_O)	16	48	0.413
MRI abnormalities	16	28	0.226
^*^post leptomeningeal metastasis; KPS: Karnofsky performance status; MRI: magnetic resonance imaging; NMNDs: nonmalignant neurological diseases; LM: leptomeningeal metastasis.			

本研究中，LM组23例(92%)因首发肺内病灶而确诊肺癌。11例(44%)确诊非小细胞肺癌(non-small cell lung cancer, NSCLC)超过3年后出现LM; 另外2例(8%，患者6和患者10)因首发神经系统症状确诊肺癌(表 2)。所有患者病程中都出现至少一个临床症状，其中15例(60%)出现头痛，14例(56%)肌力受损，10例(40%)颅神经障碍，7例(28%)痫性发作，3例(12%)后背痛，4例(16%)小便失禁。所有患者均在腰椎穿刺前行影像学检查，其中16例(64%)MRI提示异常(脑膜异常强化、结节等特异性表现)，9例(36%)MRI检查阴性(表 2)。

**表 2:** LM组患者临床资料 The clinical characteristics of LM patients

Case			Combined with BM	Times from diagnosis^*^ (yr)	Neurological symptoms	MRI abnormalities	OS after LM (mon)
Patients	Age	Sex					
1	56	F	Yes	> 3	Neck stiffness; back pain; CES	Yes	19.0
2	39	M	No	0-3	Headache; nausea; vomiting; hearing loss	Yes	9.0
3	41	F	Yes	0-3	Headache; limb weakness; CES	No	10.5
4	49	F	Yes	0-3	Headache; limb weakness; bucking	Yes	5.5
5	62	M	Yes	> 3	Ataxia; limb weakness	Yes	11.5
6	50	F	No	-	CES; memory decline	Yes	6.5
7	54	F	No	> 3	Hemiplegia; optic paralysis; bluntness	Yes	4.0
8	47	F	No	0-3	Headache; limb weakness	Yes	3.3
9	60	F	Yes	> 3	Headache; seizure; limb weakness	Yes	4.5
10	44	M	Yes	-	Bluntness; lethargy; seizure	Yes	7.5
11	55	F	No	0-3	Neck stiffness; back pain	Yes	6.0
12	51	F	Yes	0-3	Hemiplegia; headache; bluntness; seizure	Yes	7.0
13	58	M	Yes	> 3	Headache; bluntness; limb weakness	No	7.8
14	57	F	Yes	0-3	Headache; nausea; seizure; diplopia	Yes	6.3
15	50	F	Yes	0-3	Headache; memory decline; limb weakness	Yes	9.2
16	53	M	No	> 3	Headache; seizure; limb weakness	Yes	-
17	55	F	Yes	0-3	Headache; nausea; limb weakness; seizure	No	-
18	57	F	Yes	> 3	Headache; hemiplegia; bluntness; diplopia	Yes	-
19	43	M	Yes	> 3	Headache; seizure; bluntness; diplopia	Yes	-
20	60	F	No	> 3	Headache; bluntness; limb weakness	Yes	-
21	49	F	Yes	0-3	Limited vision; limb weakness	No	-
22	51	M	Yes	> 3	Headache; back pain	Yes	-
23	58	F	No	0-3	Bucking; CES	Yes	-
24	47	F	Yes	> 3	Speech disorder; headache	Yes	-
25	61	M	Yes	0-3	Headache; hearing loss	No	-
^*^LM after initial diagnosis of lung cancer; F: female; M: fale; BM: brain metastasis; OS: overall survival; CES: cauda equina syndrome.							

LM组及NMNDs组所有患者均进行了腰穿，同时评估CSF和血清相关指标。其中NMNDs组高颅压人数超过LM组(84.2% *vs* 64%)，但差异无统计学意义(*P* > 0.05)。LM组15例(60%)CSF中查见恶性肿瘤细胞，其中患者18经历两次腰穿后才获得CSF阳性。LM组血清及CSF中TM均高于NMNDs组，差异具有统计学意义(*P* < 0.05)。图 2可见NMNDs组CSF检测样本中，颅内压力值、白细胞计数、葡萄糖水平高于LM组，蛋白水平低于LM组，但差异不具有统计学意义(*P* > 0.05)，此外，NMNDs血清蛋白水平略高于LM组，差别无统计学意义(*P* > 0.05)。

**图 2:**
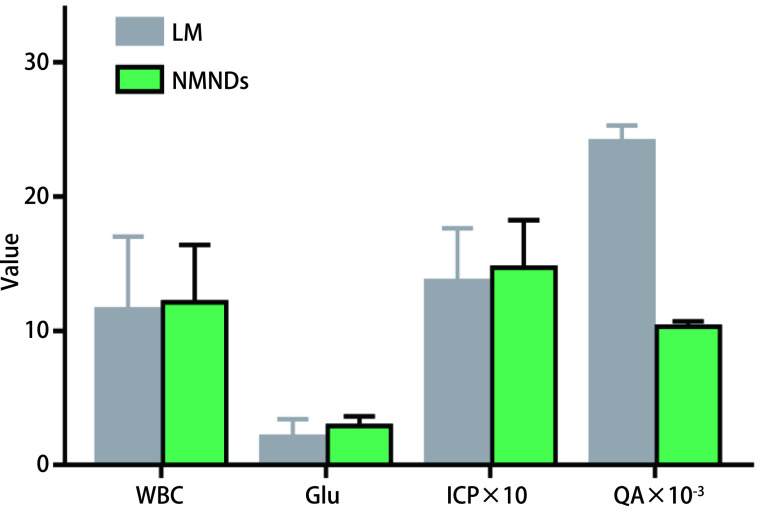
两组CSF中白细胞计数、葡萄糖水平、颅内压(80 cmH_2_O-180 cmH_2_O)和白蛋白商值对比 The comparison of white blood cell count, glucose, intracranial pressure(80 cmH_2_O-180 cmH_2_O) and quotient of albumin in CSF between two groups

### 2.2 LM组和NMNDs组血脑屏障功能评估

LM患者中94%(24/25)存在BBB障碍(QA > 8×10^-3^)，其中轻度、中度、重度和完全血脑屏障受损患者分别为：10例、12例、2例和0例(表 3)。LM组15例(60%)患者CSF中找到了恶性肿瘤细胞，其中5例(20%)CSF中血糖浓度降低(参考范围：2.5 mmol/L-4.5 mmol/L)，此外，CSF中白细胞计数在(3-52)×10^9^/L，平均在(18.9±12.7)×10^9^/L。

**表 3:** LM患者CSF中葡萄糖、QA、白细胞计数、肿瘤细胞水平 The levels of glucose, QA, white blood cells and tumor cells in CSF for LM patients

Patient	CSF glucose (mmol/L)	QA (×10^-3^)	White blood cell count (×10^6^/L)	Tumor cells (×10^6^/L)
1	4.88	12.77	12	4.0
2	5.13	26.00	3	0
3	1.89	13.41	4	0
4	4.19	28.44	15	0
5	4.95	22.00	6	4.0
6	5.29	13.85	31	17.0
7	4.46	25.00	8	3.0
8	4.99	54.00	13	25.0
9	2.03	10.30	3	0
10	4.98	26.50	17	0
11	4.09	23.00	12	5.0
12	4.67	24.02	11	3.0
13	1.01	27.47	21	20.0
14	4.86	13.11	19	4.0
15	3.59	11.53	17	0.6
16	5.09	59.00	4	30.0
17	2.58	25.00	22	0
18	3.78	21.00	9	0
19	4.89	23.00	41	5.0
20	1.95	25.00	9	7.0
21	2.45	10.10	38	0
22	4.23	13.20	23	0
23	5.90	11.20	39	0
24	5.10	9.10	49	8.0
25	2.67	6.80	52	4.0
CSF: cerebrospinal fluid.				

NMNDs组CSF细胞学均未查见恶性肿瘤细胞，其中55例(96.5%)患者BBB功能正常(QA≤7×10^-3^)，另外有2例(3.5%)CSF中检测到TM浓度高于阳性界值，余患者CSF中TM浓度在正常范围内。

### 2.3 LM组CSF中TM的鞘内合成

LM组接受检测的CSF样本中，Q TM与QA比值均大于0.7(图 3)，且Q CEA、Q CA125水平高于NMNDs组，具有统计学差异(*P* < 0.05)。LM组至少能检测到一种鞘内合成的TM(表 4)，21例(84%)鞘内合成CEA(CEA鞘内占CSF中CEA浓度：76.22%-99.99%); 22例(88%)鞘内合成CYFRA21-1(CYFRA21-1鞘内占CSF中CYFRA21-1浓度：79.24%-99.98%)，23例(92%)鞘内合成CA125(CA125鞘内占CSF中CA125浓度：82.67%-99.98%)，23例(92%)鞘内合成NSE(NSE鞘内占CSF中NSE浓度：88.55%-99.80%)。此外，LM组24例(96%)鞘内合成至少2项TM，22例患者(92%)鞘内合成至少3项TM，18例患者(72%)鞘内合成四项TM。

**图 3:**
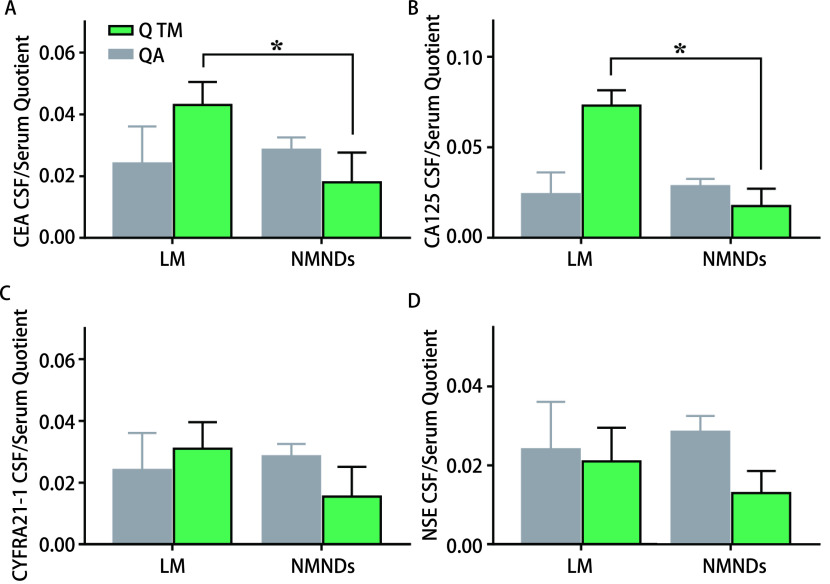
两组QA和Q TM对比(^*^P < 0.05) The comparison of QA and Q TM between two groups (^*^P < 0.05)

**表 4:** LM组患者鞘内合成TM(浓度和百分比) TM intrathecal synthesis in LM patients (concentration and percentage)

Patients	CEA_IS_			CA125_IS_			CYFRA-21_IS_			NSE_IS_	
	ng/mL	%		U/mL	%		ng/mL	%		ng/mL	%
1	15.17	76.22		98.1	99.29		4.55	97.2		48.1	99.53
2	3.16	86.04		2	90.84		4.22	98.40		13.73	94.73
3	1.35	98.20		2	98.93		6.75	97.42		7.53	98.85
4	0	0		0	0		0.77	79.24		2.81	88.55
5	65.1	99.98		56.2	99.92		4.2	99.45		15.1	99.27
6	341	98.60		12.7	98.75		0	0		13.66	98.75
7	781.74	99.48		367.6	99.61		129.8	99.95		33.17	98.42
8	1.54	95.04		116.6	82.67		3.05	96.60		15.91	96.36
9	52.19	96.92		2	84.23		1.33	99.12		6.75	97.85
10	161.3	99.60		480.2	98.65		5.33	98.98		16.56	98.58
11	78.11	99.85		9.22	97.44		161.2	93.50		35.24	95.26
12	320.12	99.98		4.11	99.36		9.75	95.45		14.53	98.71
13	0	0		0	0		1.77	90.19		0	0
14	69.1	99.99		59.2	99.98		5.2	99.81		17.1	99.80
15	398	98.95		14.7	98.91		2.78	98.91		16.66	99.04
16	789.74	99.61		369.6	99.72		149.8	99.95		39.17	98.94
17	0	0		117.6	84.73		5.05	96.95		16.91	96.49
18	69.19	93.42		5.99	86.34		3.33	98.07		8.75	95.47
19	178.4	99.03		489.2	96.65		8.33	97.84		18.56	96.62
20	3.21	96.82		14	95.85		0	0		0	0
21	4.21	91.31		3.7	88.32		3.22	95.6		3.89	89.12
22	5.24	88.34		0	0		4.12	97.2		2.66	90.21
23	0	0		2.84	85.29		2.78	89.1		1.68	94.26
24	2.44	82.34		3.18	92.11		5.26	88.9		1.06	93.22
25	1.67	85.92		4.25	90.25		7.88	97.2		2.05	97.49
IS: Intrathecal synthesis; CEA: carbohydrate antigen; CA125: carbohydrate antigen125; CYFRA-21: cytokeratin 19 fragments; NSE: neurone specific enolase.												

1例患者(患者18)，在首次腰椎穿刺后CSF细胞学检查阴性，但检查提示有鞘内合成TM(CEA鞘内：69.19 ng/mL，占CSF浓度93.42%;CA125鞘内：5.99 U/mL，占CSF浓度86.34%;CYFRA21-1鞘内：3.33 ng/mL，占CSF浓度98.07%;NSE鞘内：8.75 ng/mL，占CSF浓度95.47%)，对该患者进行第二次腰穿时，CSF细胞学阳性，最终明确了LM诊断。

### 2.4 LM组CSF中TM检出情况与鞘内合成相关性分析

本研究中所有患者行周期性脑室内化疗，平均治疗4.5个周期，其中64%(16/25)的CSF中TM阳性，CEA阳性在CSF中最常见，占68.8%(11/16)，其中CEA单独阳性共5例，CEA联合余三项TM中任一项为阳性共8例，CSF中CEA阴性而余三项中任一项TM阳性共5例。

临床工作中，常常以CSF中TM浓度辅助诊断LM，将CSF中TM指数与TM检出阳性率2种方法进行对比，使用线性加权Weighted Kappa系数分析，评估TM指数与TM检出阳性率对LM诊断是否具有相关性，结果显示，CEA指数与检出阳性率对25例LM患者诊断的阳性一致率为88.9%，阴性一致率为18.2%，一致性检测Kappa值为0.065;CA125指数与检出阳性率诊断的阳性一致率为91.7%，阴性一致率为12.5%，Kappa值0.071;CYFRA21-1指数与检出阳性率诊断的阳性一致率为76.9%，阴性一致率为0%，Kappa值为0.06;NSE指数与检出阳性率诊断的阳性一致率为66.7%，阴性一致率为5.9%，Kappa值为0.09。总的来说，CSF中TM指数与TM阳性界值对LM诊断结果具有不一致性，且无统计学差异(*P* > 0.05，表 5)。

**表 5:** LM组TM指数与CSF中TM检出率的一致性分析 The consistency analysis between TM index and the positive rates of CSF TM in LM group

Item	PCR (%)	NCR (%)	Kappa	*P*	95%CI
CEA	88.9	18.2	0.065	0.212	-0.220-0.351
CA125	91.7	12.5	0.071	0.389	-0.039-0.182
CYFRA21-1	76.9	0	0.06	0.43	-0.032-0.153
NSE	66.7	5.9	0.09	0.144	-0.296-0.117
PCR: positive consistency rate; NCR: negative consistency rate.					

### 2.5 动态监测CSF鞘内合成TM的临床价值

所有患者每次行脑室内化疗前留取同期CSF和血清，计算和记录CSF中鞘内生成TM、细胞数和QA。其中患者1在治疗6个月后，CSF细胞数变化与白蛋白商值、鞘内合成CEA的变化相似，该患者超过2年保持临床稳定(图 4)。

**图 4:**
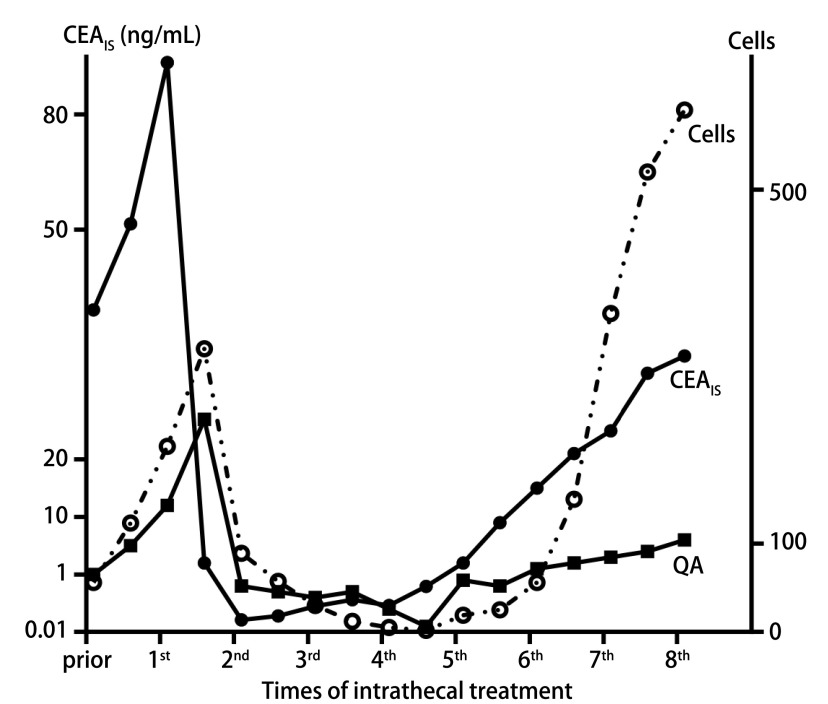
1例LM患者经Ommaya囊脑室内化疗过程中，鞘内合成CEA，CSF蛋白与血清蛋白比率以及CSF肿瘤细胞数的动态变化 Intrathecal synthesis CEA fraction (CEAlS), CSF/serum quotient of albumin (QA) and CSF tumor cells in a case of LM patients under intrathecal chemotherapy via Ommaya Reservoirs

## 3 讨论

LM是晚期肺癌致残和致死的重要原因之一，预后极差^[14]^。CSF细胞学是诊断LM的基石，但诊断敏感性低，尤其是在LM早期，导致治疗时机的贻误^[1, 6, 15]^。因此，在CSF中寻找诊断LM敏感性高的指标至关重要。CSF中TM浓度的升高常常被用于LM的辅助诊断^[16]^，CSF中TM浓度升高取决于：①局部癌细胞分泌释放; ②血清中TM通过BBB渗入，前一种可能性更大^[17]^。CSF中TM的诊断LM敏感性低，尤其是在LM疾病早期，临床应用受限，很大程度上因为CSF中TM浓度取决于血清水平和BBB状态，因此，相较于检测TM浓度，判断CSF中TM的来源对LM诊断同样具有重要诊断作用。

我们前期通过CSF中TM浓度诊断LM的研究中，发现部分患者血清TM正常，而CSF中TM升高; 也存在患者血清TM和CSF中TM同时升高的现象^[18]^，提示LM存在鞘内TM合成。目前对于CSF中TM升高影响因素的相关研究较少。Schold等^[19]^发现血清CEA浓度升高的卒中患者，BBB破坏后CSF中CEA未见升高。同样有文献^[9]^报道，除非血清TM浓度足够高，且血清与CSF中CEA浓度比值超过60:1，才能透过BBB渗入CSF中，影响CSF中TM浓度。有文献^[9, 11, 20, 21]^报道提出，TM和IgG均为小分子物质，分子量相近，血清中TM遵循与IgG滤过BBB相似的扩散定律，可通过IgG指数公式计算鞘内CEA合成，明确鞘内CEA来源对CNS疾病进行诊断分类。Kang等^[9]^分析CEA商值(CSF与血清CEA比值)与白蛋白商值具有相关性，排除BBB损伤对CSF中CEA浓度的干扰，更好地评估CSF中CEA升高与癌细胞分泌相关，诊断特异性强。CSF与血清CEA比值正常或者未检测到，可能是颅内癌细胞未分泌CEA，并不能完全排除LM，且相关研究入组的是多种实体瘤伴LM(乳腺癌、肠癌、肺癌等)，诊断敏感性不高。因此，本研究入组的均为肺癌伴LM，选择对肺癌诊断敏感性高的4项肿瘤指标，通过分析QA评估BBB的完整性、CSF与血清肺癌4项比值判断CSF中TM来源，评估LM患者颅内TM升高的影响因素，希望为肺癌LM的诊断提供一种全新的诊断方法。

本研究中，利用CNS疾病诊断中重要的计算公式，即IgG指数，分析比较LM组与对照组鞘内TM合成的差异，评估鞘内TM合成对LM诊断的敏感性和特异性，为LM的临床诊断和鉴别诊断提供依据。本研究数据显示，鞘内合成的CEA、CA125、CYFRA21-1、NSE对LM的诊断敏感性分别为84%、88%、92%、92%，特异性高达100%，对照组CSF中未发现鞘内合成TM，而LM组所有CSF中均发现有鞘内TM合成。另外，LM组所有患者至少存在一种鞘内TM合成，因此，对疑似LM患者行CSF检测时，可同时进行CSF中CEA、CA125、CYFRA21-1、NSE的检测，计算4种TM鞘内合成，通过判断CSF中TM来源诊断LM。研究还发现，CSF中TM检测浓度超过76.22%-99.98%来源于鞘内合成，且鞘内TM合成与CSF中TM检出情况不一致，因此，对所有行CSF中TM检测的患者分析鞘内合成并定量，明确CSF中TM来源有助于LM的诊断，并成为LM诊断敏感性高和特异性强的有效工具。

本研究中，所有患者在经Ommaya脑室内化疗前收集脑室内CSF动态监测TM。有趣的是，患者1在诊断LM时提示有3种TM的鞘内合成，经过脑室内化疗2个周期后症状明显缓解，CSF中TM浓度、鞘内合成的TM、CSF细胞数明显下降，治疗6个月后CSF中细胞学转阴，最终该患者在LM后获得了2年生存期。由此推测，CSF中鞘内合成的TM或许可作为评价LM治疗疗效的指标之一，接受脑室内化疗患者CSF中CEA的变化早于CSF细胞学变化，更早地提示治疗疗效和预警复发，对于有鞘内合成TM的LM患者而言，连续动态监测TM可能具有临床应用价值，但是因为病例数少，这个单一的发现并不能作为结论，期待后续有更多的研究数据支持TM潜在的应用价值。

文献^[22]^报道，CSF细胞学阳性LM患者中50%细胞计数正常。本研究中，LM组60%患者CSF中均检测到了癌细胞，8例(32%)CSF中细胞计数正常，这与文献报道一致，提示临床工作中CSF细胞计数正常，也不能完全排除LM，CSF细胞学检测应始终作为常规检查，一旦怀疑有恶性细胞，应对所有CSF标本仔细行细胞形态学分析。在CSF细胞学阴性的情况下，90%的LM患者观察到CSF间接变化，包括颅内压升高(> 200 mmH_2_O)占21%-42%，白细胞计数增加(> 4 mm^-3^)占48%-77%，蛋白质升高(> 50 mg/dL)占56%-91%，葡萄糖降低(< 60 mg/dL)占22%-63%^[23]^。临床上，CSF细胞学阴性但白细胞计数升高的现象，对鉴别是癌性脑膜炎，还是感染或免疫疾病导致CSF变化带来了极大困难，尤其是结核性脑膜炎，无论是临床症状/体征，还是CSF异常均与LM有诸多相似^[24]^，难以鉴别; 另外肿瘤患者发生LM前往往接受过多线放化疗，机体处于免疫抑制状态，更易出现CNS感染，如真菌或者其他机会性病原体感染，以上种种原因都会出现颅高压症状、CSF检查异常^[25]^，从而导致LM误诊和漏诊，耽误治疗时机，因此，对于首次检测CSF细胞学阴性，TM检测以及鞘内合成TM计算可进一步提高诊断的敏感性，必要时重复腰穿行细胞学检查，从而避免治疗时机贻误。CSF中葡萄糖浓度低可见于多种感染性疾病，而LM发生BBB损伤，葡萄糖膜转运受损，血清中葡萄糖向CSF有效运输降低，也可导致葡萄糖水平下降。据文献^[26]^报道，细菌性脑膜炎患者CSF中葡萄糖浓度低于1 mmol/L，而本研究中20例(80%)LM患者CSF中葡萄糖水平正常，表明CSF中葡萄糖水平对LM的诊断价值有限。

当然，本研究还存在许多不足，该研究为一项单中心、小样本回顾性研究，对这25例LM患者观察结果是初步的，还需要纳入更多患者进一步评估并确认这些发现，以明确CSF中TM的诊断和预后价值。另外，本研究纳入肺腺癌人群，无法探究鞘内不同TM合成是否与肺癌不同组织类型相关。本研究首次纳入肺癌来源的LM患者，检测对肺癌敏感性高的4项TM，尝试QA和IgG指数公式，评价BBB完整性，判断鞘内TM来源并使之成为LM诊断的一个特定而敏感的参数，动态观察BBB完整性及鞘内TM合成量的演变规律，也有助于临床判断病情、疗效及预后的评估。
